# High precision and real-time acquisition system for interface stress measurement in bridge bearing

**DOI:** 10.1038/s41598-023-28848-x

**Published:** 2023-01-28

**Authors:** Xinning Cao, Guangming Li, Zhe Li, Wujie Sun, Fabao Yan, Ruijuan Jiang

**Affiliations:** 1 School of Mechanical, Electrical and Information Engineering, Shandong University, Weihai, 264209 China; 2 North Automatic Control Technology Institute, Taiyuan, 30006 China; 3 Shenzhen Municipal Design and Research Institute Co., Ltd., Shenzhen, 518029 China; 4 Weillai Research Institute of Industrial Technology of Shandong University, Weihai, 264209 China

**Keywords:** Electrical and electronic engineering, Civil engineering

## Abstract

Since the damage of the bridge structure may cause great disasters, it is necessary to monitor its health status, especially the bridge bearing, the important connecting component of the bridge's upper and lower structures. Nowadays, manual inspection is the main method to get the information of the bridge bearings’ work status. However, occasional damage of bridge bearing may not be detected in time, and sometime the installation position of the bearing makes the manual inspection on bridge bearing difficult and even impossible. Therefore, in order to know the work status of the bridge bearings timely, an intelligent remote monitoring system for the bridge bearing is developed. A 32-channel real-time acquisition system is designed by using an AD7768-1 analog-to-digital converter and Xilinx Spartan-6 Field Programmable Gate Array for interface stress continuously acquired in the bridge bearing. To assure the good linearity and low noise performance of the monitoring system, the data acquisition card is meticulously designed to reduce noise from both hardware and software and realize high-precision acquisition. Through the establishment of the monitoring server, the compressive stress data can be displayed synchronously and the overpressure situation can be alarmed in real-time. The experimental results show that the accuracy of the calibrated sensor is within 1.6%, and the detection error of the acquisition board is less than 200 μV. The acquisition system is deemed to have considerable advantages in accuracy and applicability.

## Introduction

Wide application of bridges makes transportation more convenient. However, there may exist a series of potential serious problems, for example, impact on the bridge structure, long-term overload, environmental factors, etc.^1^. Due to the above problems, many bridges around the world collapsed, such as the Ezhou huhua Interchange Ramp^2^, Morandi Bridge in Italy^3^ and Florida International University pedestrian bridge^4^, etc. Those collapses of bridges caused serious disastrous disasters to people's lives and the social economy, which prompted people to hope for a system with a comprehensive and long-lasting health monitoring on the bridge. The structural health monitoring (SHM) system of bridges emerged to meet this demand^5^.

In recent years, SHM has been widely used around the world for operational and building phase, including the Golden Gate Bridge^6^ in the United States, Tamar Bridge^7^ and Humber Bridge^8^ in the United Kingdom, Yokohama Bay Bridge^9^ etc. A typical SHM system usually consists of four parts: sensors, a data acquisition system, a data transmission system and a state evaluation system^10^. With the development of intelligent technology in various fields, bridge monitoring enters a turning period of full intelligence and continuous improvement of monitoring accuracy. Among them, the most critical point of accurate monitoring of bridge health condition is data, mainly for the improvement and development of three aspects: bridge sensor, data acquisition board, noise reduction algorithm optimization.

Cao et al.^11^ developed a strain force sensor with double cantilever beam structure for solving the problem of low efficiency of bridge disturbance measurement. According to the experiments, it is known that the sensor has good linearity and repeatability with a maximum linearity error of 0.25% and repeatability error of 0.28%, but the cost of the sensor is high. M. Pieraccini et al.^12^ developed Interferometric multiple-input multiple-output (MIMO) radar for remote monitoring of bridges, which has been successfully tested as geotechnical equipment during the real case of the monitoring of a historical bridge in the city of Florence, Italy. Maha Sliti et al.^13^ pointed out that a fiber Bragg grating sensor can improve the structural safety through real-time vibration monitoring, which means that the Fiber-optic Bragg grating sensor has great potential for structural monitoring. Sung-Jin and Nam-Sik developed a fiber-optic grating-based sensor, but this sensor causes significant changes in the support structure and affects the reliability of the mechanical structure^14,15^. In the current variety of sensors, the selection of sensors does not only need a high index of performance. According to the actual demand of bridge monitoring, the technical indexes and procurement cost should be considered, and the sensor suitable for the designed bridge monitoring system should be selected.

Cui et al.^16^ used a general-purpose acquisition card with 16-bit resolution to obtain accurate data in order to achieve real-time diagnosis of the health condition of the Tieluoping Bridge, but the cost of the general-purpose acquisition card is too high for large-scale use. Chen^17^ used two Analog-to-digital converters to realize synchronous sampling of 12 channels. They have increased the integration of the acquisition cards by increasing the number of acquisition signal channels, which effectively reduced the number of data acquisition cards used in the system. However, the general high-performance acquisition board has fewer input ports and high cost, which is limited for engineering batch application. According to the characteristics of the bridge health monitoring parameters, the design of high-precision special acquisition board, which integrates cost and performance, is more suitable for bridge monitoring system.

Noise reduction algorithm optimization can perform noise reduction processing based on signal characteristics, and secondary processing of noise that cannot be removed by hardware circuits. With the development of artificial intelligence algorithms, algorithm optimization has been applied in bridge monitoring systems. Nguyen^18^ analyzed the effect of vehicle movement on bridge cracks by wavelet spectrum, and the location of breathing cracks can be accurately located at 10% noise. Jin et al.^19^ trained the extended Kalman filter on the neural network and applied it to the detection of bridge damage by temperature difference. The average error of their detection is about 1.1%, which improves the measurement accuracy. According to the noise reduction processing of different monitoring parameters, it is very important to select the algorithm suitable for the characteristics of the monitoring signal.

In the data transmission system, the traditional wired communication has reached maturity in terms of information transmission stability and information carrying capacity. The development of wireless communication technology has expanded the information communication method and provided new ideas for bridge monitoring information communication. Whelan et al.^20^ introduced wireless sensing technology into bridge vibration testing. Nie et al.^21^ proposed a bridge monitoring architecture through ZigBee. Al-Radaideh et al.^22^ applied ZigBee transmission and GPRS transmission to bridge data transmission system at the same time. However, during the data transmission process of the wireless communication method, the signal is susceptible to electromagnetic interference, which means that the stability and security of this type of data transmission are poor. People have been pursuing high-precision, multichannel, and wireless communication methods in bridge monitoring systems. Progress in these aspects has contributed to a better monitoring of bridge status. But the acquisition board with high-performance has fewer input ports and higher price, which causes limitations for engineering batch applications. Monotonic transmission is difficult to ensure data stability and efficiency in long-term bridge monitoring.

In view of the development status of bridge intelligent monitoring system, higher monitoring accuracy, more data collection and diversified transmission methods have been the development trend of bridge monitoring system. The system's multichannel acquisition makes it possible to arrange dense test points, which is a prerequisite for comprehensive and accurate bridge monitoring. The system's high-precision detection grasps the bridge health condition more precisely, which is the key point of designing the bridge monitoring system. Multiple communication methods break the limitation of single data transmission method, which is the necessary condition for flexible and accurate data transmission of the monitoring system. According to the above characteristics, we develop a high-precision and real-time data acquisition system to measure the interface stress of the bridge support. In order to meet the demand for bridge bearing monitoring, we design a monitoring system with high accuracy, multi-channel and multiple communication methods, and reduce the use of hardware resources and system cost while ensuring the applicability of the bridge bearing monitoring system. For the characteristics of bridge bearing compressive stress, the average weighting operation and the traceless Kalman filter algorithm are used to process the collected data for primary and secondary noise reduction respectively, which improves the monitoring accuracy of the system.

The rest of this paper is organized as follows. In Section "Materials and methods", the overall design of the system is given. In Section "System hardware design", the sensor module, signal conditioning module, ADC module, FPGA module and communication module are introduced. In Section "Software system design", the internal logic of FPGA, unscented Kalman filter and the construction of monitoring server are introduced. Experimental results of the proposed system are presented in Section "Test and result". Conclusions and remarks on possible further work are given finally in Section "Conclusion".

## Materials and methods

**Figure 1:**
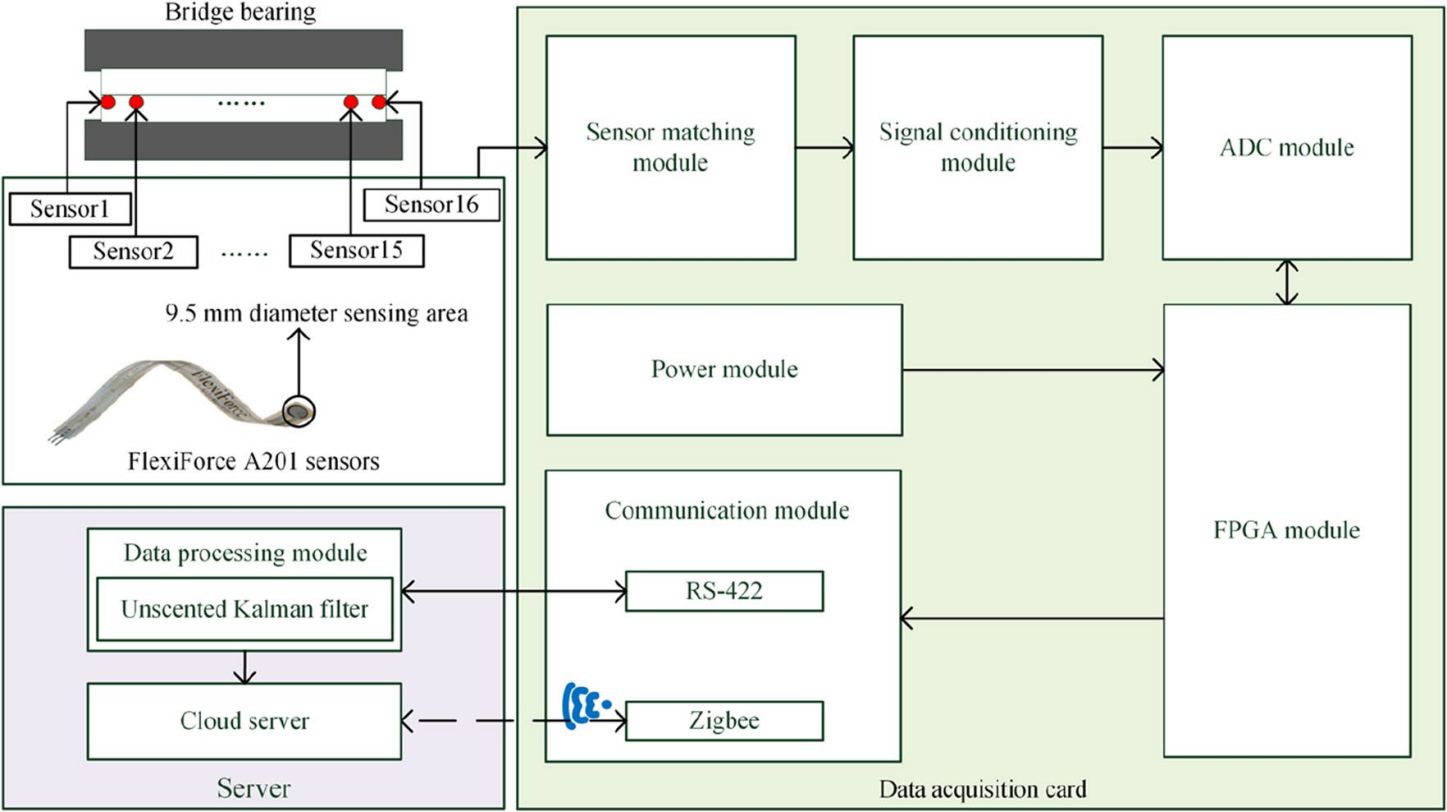
Block diagram of bridge support monitoring system.

According to the bridge speed limit requirements and vehicle body design standards, the signal frequency collected by the bridge bearing monitoring system is less than 100 Hz^23^, so the system can collect signals within 100 Hz, and adjustable within 1 ~ 100 Hz. The monitoring system contains three parts: sensor, data acquisition card, communication system and server. Fig. 1 shows the principal block diagram of the bridge support monitoring system.

Sensor is the device to get the measurements. Its performance directly affects the monitoring accuracy of the whole system^24^. The system proposed in this paper uses FlexiForce piezoresistive pressure sensors to measure the interface stresses. The sensors are flexible and each has a 9.5 mm diameter sensing area, limiting the influence of the sensing system on the bridge bearing, while keeping a high-resolution measurement capability.

The sensor transmits the collected signals to the data acquisition card for processing. The physical signal measured by the sensor is converted into an analog voltage signal by the Sensor matching module, which is convenient for the lower circuit to process the signal. Signal conditioning module amplifies and filters the obtained analog voltage signal. ADC module converts analog voltage signals into digital signals. In order to reduce the influence of quantization error on accuracy, we use 24-bit high-resolution ADC. The acquisition system for the bridge bearing requires multi-channel synchronous acquisition, fast acquisition speed and high accuracy. The parallel processing capability of the FPGA allows each interface to be operated independently, increasing the speed of data control and acquisition. And the internal logic can be reconfigured according to requirements, reducing development costs. In addition, for long-term maintenance, FPGA chips can be field upgradable. As the system matures, users can directly program it without having to redesign the hardware circuitry to add new features. Therefore, the core control module of this design uses FPGA microprocessor. The communication system includes wired and wireless communication. Recommended Standard 422 (RS-422) and Zigbee are used for wired communication and wireless communication, respectively. The combination of two communication modes avoids the limitation of a single communication mode.

The bridge site server processes the data transmitted by RS-422 and sends the valid data to the cloud server. Zigbee directly establishes communication with the cloud server to realize wireless transmission. The stress data is stored in the database orderly, which is convenient for managers to monitor in real time.

## System hardware design

**Figure 2:**
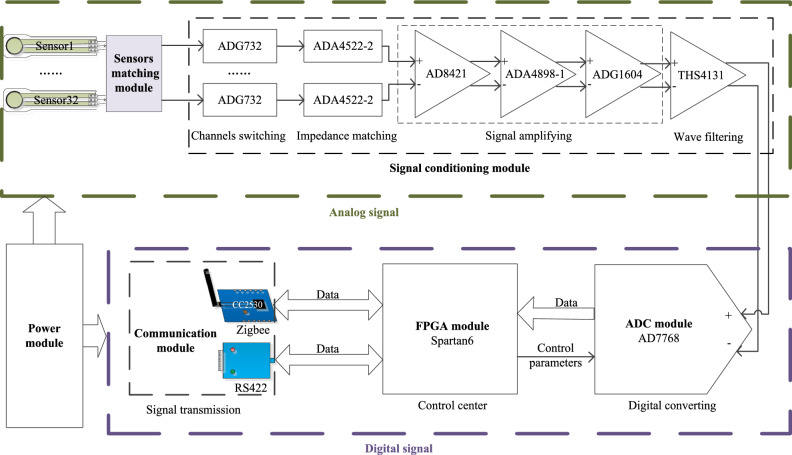
Block diagram of data acquisition card.

As shown in Fig. 2, the data acquisition card includes sensor matching circuit, signal conditioning circuit and digital circuit. The sensor matching circuit converts the measured impedance into an analog voltage signal. The signal conditioning circuit includes channel selection, impedance matching, signal amplification circuit and filter circuit. The signal conditioning module converts the signal into an analog signal that can be easily processed by the ADC, and then converts it into a digital signal through the ADC module and inputs it into the FPGA before transmitting it to the server via RS-422 or Zigbee communication.

### Sensor module

#### Sensor selection

The compressive stress borne by the bridge bearing has a wide range of amplitude and low frequency. And the selected sensor needs to meet the requirement of repeated strain. In addition, the precision of sensor is one of the important factors of high-precision monitoring. At present, there are four kinds of sensors suitable for bridge bearing monitoring. Their performance specifications are shown in Table 1.

**Table 1:** Performance indicators for different types of sensors.

Sensor name	Precision	Force measurement range	Response time	Repeatability
Resistance strain sensor	0.1%FS	0–10^3^N	5 $\mu {\text{s}}$	± 2.5%^25^
Grating strain sensor	1%FS	0–10^4^N	70 ns	± 0.1%^26^
Hydraulic sensor	0.25%FS	0–10^7^N	10 ms	− ^27^
Piezoresistive film sensor	0.25%FS	− 0.1–10^7^N	10 ms	− ^28^

Resistance strain sensor is widely used, but its measuring range is narrow, and it is easily affected by temperature. Thus, it cannot accurately measure the compressive stress of bridge bearing. Grating sensor has strong anti-interference ability, but its manufacturing process is complicated and its price is high. So, it is not suitable for multi-point monitoring. Although the hydraulic sensor has a wide force measuring range and high precision, it cannot be replaced after installation. Considering its high cost, it is not suitable for long-term monitoring of bridge supports. Piezoresistive thin-film sensors have zero drift, but are easily compensated for in monitoring systems. Due to the low signal frequency of bridge bracket monitoring, the selected sensor does not need to be excessively sensitive, but high accuracy is required. the advantage of FlexiForce A201 is its high accuracy and wide measurement range, which can ensure high precision compressive stress monitoring of bridge brackets. Therefore, the FlexiForce A201 piezoresistive thin-film sensor was selected to measure the compressive stress. The performance specifications of FlexiForce A201 are shown in Table 2.

**Table 2:** FlexiForce A201 performance indicators.

Pressure-bearing capacity	Linearity	Repeatability	Hysteresis	Drifting	Response time
0 ~ 445N	± 3%	± 2.5%	< 4.5%	< 5%	< 5 $\mu {\text{s}}$

#### Sensor matching circuit

**Figure 3:**
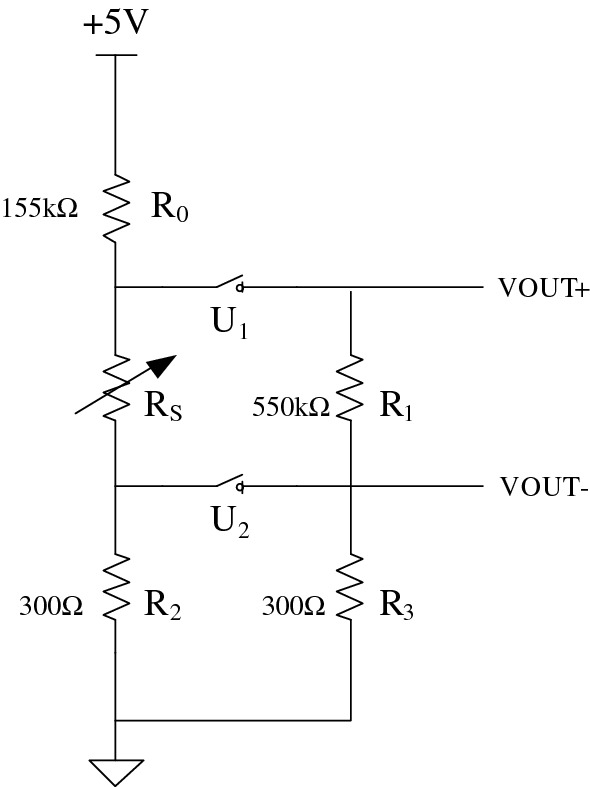
Equivalent circuit with analog switch peripheral circuit.

According to the range of compressive stress measured by the support, the resistance value of FlexiForce A201 sensor is preliminarily selected to be between 0 and 550kΩ. 32 channels are used for data transmission. If 32 channels are used for signal acquisition at the same time, a lot of resources will be consumed. To solve this problem, two analog switches ADG732 is used to realize time division multiplexing and collect signal from 32 channels. Fig. 3 shows the equivalent circuit diagram of one sensor and its periphery.

When U_1_, U_2_ are selected, according to Ohm's theorem, the relationship between the output voltage (V_out_) and the sensor resistance (R_s_) can be known as shown in Eq. (1).

1 $$ \frac{{V{\text{out}}}}{RS//R1} = \frac{ + 5V}{{R0 + RS//R1 + R2//R3}} $$

Simplifying it, we get as shown in Eq. (2).

2 $$ \frac{1}{RS} = \frac{1}{R0 + R2//R3} \times \left( {\frac{Vcc}{{Vout}} - 1} \right) - \frac{1}{R1} $$

This circuit is powered by 5 V reference. R_s_ is the equivalent resistance of the sensor with the maximum value of 550kΩ. U_1_ and U_2_ are ADG732. R_1_ and R_3_ are the output resistances of ADG732. When U_1_ and U_2_ are gated, according to the ADG732 data sheet, the recommended resistance R_3_ is 300Ω^29^. So R_2_ is 300Ω. According to the maximum R_s_ value of 550kΩ, R_1_ equals to 550 KΩ, and then R_0_ equals to 155kΩ.

### Signal conditioning module

In order to realize accurate monitoring, the signal conditioning module is subdivided into front-end circuit, first-stage amplifier circuit, second-stage amplifier circuit and low-pass filter circuit.

The sensor signal is weak at the beginning. The resistance is the same order of magnitude as the input impedance of the lower-level instrumentation amplifier. This may cause the impedance mismatch between the sensor and the amplifier. In order to solve this problem, ADA4522-2 is selected as an operational amplifier to amplify the impedance. The operational amplifier is connected to the two output ends of the differential mode signal to achieve impedance matching. In order to avoid the interference of high frequency noise, a radio frequency interference (RFI) filter is designed at the output end of the operational amplifier, which can suppress the high frequency signals of the common and differential modes at the same time.

After RFI filtering, the signal is weak. And it is easy to introduce noise, which increases the difficulty of the next signal processing circuit and even the signal get lost. Thus, AD8421 and ADA4898-1 are used to enhance RFI filtering and differential amplification of signals. The AD8421 is a high-speed instrumentation amplifier with low cost, low power and low noise. The ADA4898-1 is a high-speed voltage feedback amplifier with stable unity gain and low noise.

Because the bearing compressive stress of bridges with different spans is different, a secondary amplifier circuit is set up to facilitate the acquisition of signals with different amplitude ranges. ADG1604 is selected as a complementary metal-oxide semiconductor (CMOS) analog multiplexer. ADA4841-2 is selected as a gain stable, low noise and distortion, rail-to-rail output amplifier to achieve the function of signal amplification with adjustable magnification.

Because the sensor analog signal is doped with noise during the processing, the amplifier circuit amplifies the useful signal and also amplifies the noise. Thus, we design a second-order Butterworth low pass filter made of THS4131. Since the bridge bearing monitoring signal frequency is less than 100 Hz, the -3 dB bandwidth is set to 100 Hz.

**Figure 4:**
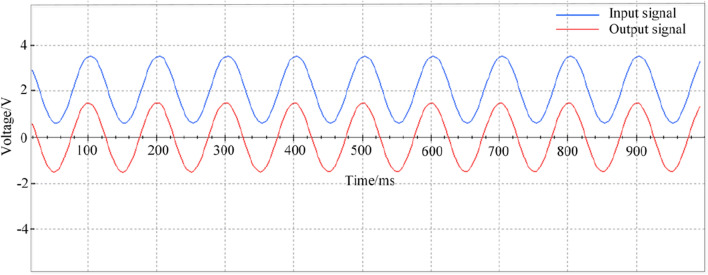
The input signal and output signal diagram of the signal conditioning module.

The input and output signals of the signal conditioning circuit have been tested, as shown in Fig. 4.

Channel A of the top signal represents the input signal, and channel B of the bottom signal represents the output signal of the signal conditioning module. The test chart shows that the signal conditioning circuit can maintain the original signal characteristics well.

### ADC module

The compressive stress of bridge supports is processed by signal conversion, signal amplification, filtering, etc. The physical signals collected by sensors are converted into analog signals which are easy to be processed by ADC^30,31^. Accurate ADC conversion can reflect the physical information of the collected signal, so it is very important to select an ADC with appropriate performance. The signal frequency collected by the bridge bearing monitoring system is less than 100 Hz. With combined consideration of ADC sampling frequency, linear error analysis of thin film sensor and chip cost, AD7768-1 chip is selected as the analog-to-digital converter for compressive stress monitoring of bridge support.

The AD7768-1 is a Σ-Δ analog-to-digital converter with low power and high performance. Because the serial peripheral interface (SPI) four-wire mode interface is simple and easy to configure the data conversion mode, we adopt the SPI control mode of the ADC in this system. To avoid the impact of power supply ripple in the process of data acquisition, the low-noise reference sources are used to power the ADC. To avoid the signals errors due to different reference sources, the common-mode voltage of the AD7768-1 analog-to-digital converter is taken as the reserve bias reference for the front-end conditioning amplifier. This may further improve the accuracy of the analog-to-digital conversion.

### FPGA module

With the increasing processing power of FPGA chips, they are widely used in the fields of signal acquisition and data processing, etc. Major FPGA suppliers such as Xilinx, Altera, etc. develop chips with different performance. By comparison, Xilinx FPGAs have richer shorting resources and higher wiring success rate, and Altera FPGAs have larger RAM modules. Synthetically consider the comprehensive cost and the performance of the monitoring system, Xilinx Spartan-6 series FPGAs are finally selected.

The FPGA is configured into active parallel mode through two mode pins M [1:0]. After power-on, the configuration data bit stream is automatically loaded from Programmable read-only memory (PROM) to the internal static random-access memory (SRAM) of the FPGA. An 8-bit data bus width and the configuration clock provided by the FPGA are used to configure the active parallel mode, and reserve the JTAG configuration expansion port to facilitate debugging of the FPGA.

### Communication module

To improve the flexibility of system communication, the bridge bearing interface stress monitoring system integrates multiple data transmission methods. The data tran smission methods used in this design include RS-422 and industrial ZigBee communication. RS-422 has a long communication distance and is suitable for long-distance data transmission, but its number of nodes is limited. Zigbee can control 256 nodes in each coordinator, but its transmission distance is limited. The system adopts a combination of wired and wireless data transmission, and the communication emergency program is switched through the upper computer, giving priority to wireless transmission. The program internally judges at regular intervals whether the data stored in the database for wireless transmission is normal, and if it is not normal, the wired communication service is switched immediately. This ensures real-time data monitoring.

## Software system design

**Figure 5:**
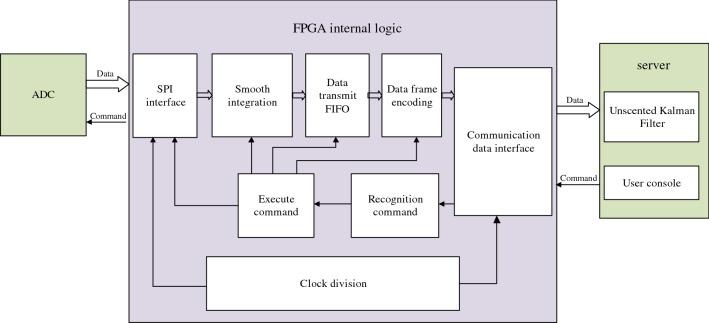
Monitoring system software design block diagram.

The software design of bridge bearing monitoring system mainly includes FPGA data processing, server secondary data processing and monitoring server. The system block diagram is shown in Fig. 5.

The selection of ADC acquisition mode, the weighting times of smoothed data and the reasonable configuration of FIFO depth capacity can realize the self-adaptation of accurate measurement of the system. Then, the server performs secondary processing on the collected data to further improve the accuracy of data. In addition, the stress monitoring server is designed to realize the real-time communication between the bridge site server, cloud server and data acquisition system, with the functions of data storage and real-time alarm.

### FPGA internal data processing

**Figure 6:**
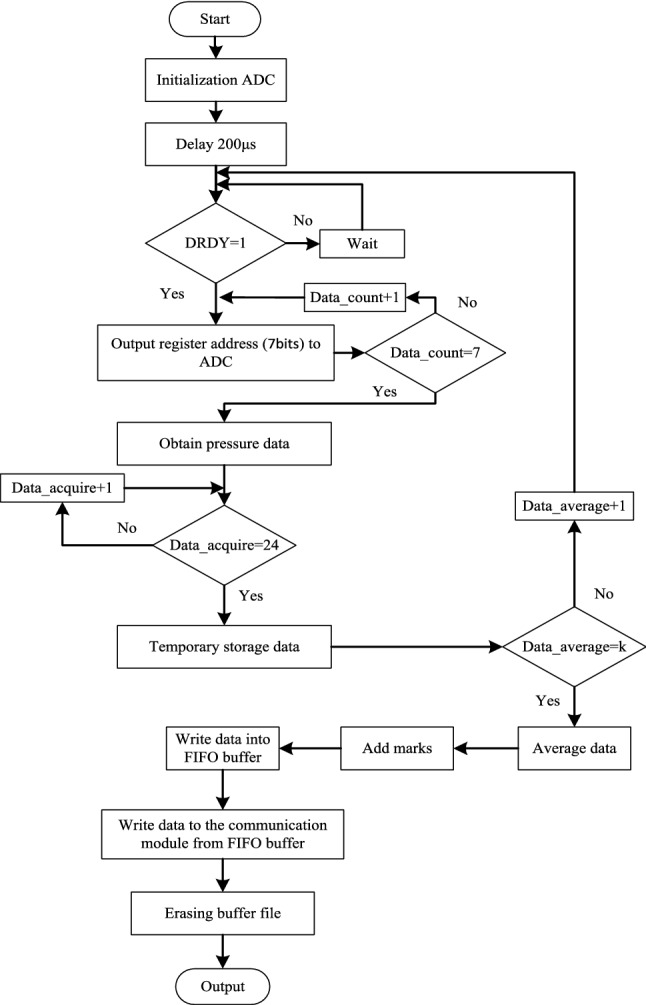
The flowchart of FPGA internal logic development.

Figure 6 illustrates the flowchart of FPGA internal logic development. The flow chart shows the configuration methods of ADC, data smoothing and so on.

After the system is powered on, it will generate an automatic reset signal. When the controller receives the reset signal, it will initialize the ADC chip. The objects of ADC initialization mainly include ADC chip selection signal, read–write mode, power mode, frequency division coefficient and decimation rate, etc. It will delay 200 μm to ensure that ADC initialization is completed. When FPGA receives the low-level DRDY signal, FPGA accesses the data storage register to obtain the collected compressive stress value. The output 24-bit data will be averaged. It assigns the processed compressive stress value to each channel. And outputs the data of each channel through channel switching.

### Unscented kalman filter

Because FPGA has limited processing ability for complex algorithms, unscented Kalman filter algorithm is added to the upper computer for secondary processing data. This algorithm mainly builds a mathematical model according to the characteristics of the monitoring signal, and realizes the noise reduction during signal processing.

According to Eq. (2), the relationship between the bearing compressive stress resistance *R*_*i*_ and the measured voltage *Z*_*i*_ grams expressed as shown in Eq. (3).

3 $$ Zi = \frac{1}{{\frac{31030}{{Ri}}{ + }\frac{43761609}{{170665000}}}} $$

According to the relationship between the state variables of the nonlinear system and the observed variables, the relationship between the bearing compressive stress *X*_*i*_ and the corresponding measured voltage *Z*_*i*_ can be known. Since the correspondence between the bearing compressive stress *X*_*i*_ and the resistance *R*_*i*_ is determined by the calibration of the sensor, here, for the convenience of discussion, the linear relationship according to the parameters of the sensor is taken as a quadratic fitting relationship, as shown in Eq. (4).

4 $$Ri = \frac{30 \times 31030}{{\sqrt {Xi} }}$$

According to Eq. (3) and Eq. (4), the relationship between the bearing compressive stress X and the corresponding measured voltage Z can be approximated as shown in Eq. (5).

5 $$Zi = \frac{1}{{\frac{{\sqrt {Xi}}}{30} + \frac{10}{{39}}}}$$

An *n*-dimensional random state variable with known mean $\overline{X}$ and covariance *P* can be computed as a set of 2*n* + 1 sigma points by a traceless transformation. As shown in Eq. (6).

6 $$ X^{(k)} = \left\{ {\begin{array}{*{20}l} {\overline{X} } \hfill \\ {\overline{X} + (\sqrt {(n + \lambda )P} )_{k} } \hfill \\ {\overline{X} - (\sqrt {(n + \lambda )P} )_{k} } \hfill \\ \end{array} \begin{array}{*{20}l} {,k = 0} \hfill \\ {,k = 1\sim n} \hfill \\ {,k = n + 1\sim 2n} \hfill \\ \end{array} } \right. $$

The weights of the 2*n* + 1 sigma points corresponding to the mean and covariance, respectively, are shown in Eq. (7).

7 $$ \omega_{s}^{(k)} = \left\{ {\begin{array}{*{20}l} {\frac{\lambda }{n + \lambda }} \hfill \\ {\frac{1}{2(n + \lambda )}} \hfill \\ \end{array} } \right.\begin{array}{*{20}l} {,k = 0} \hfill \\ {,k = 1\sim 2n} \hfill \\ \end{array} \quad \omega_{c}^{(k)} = \left\{ {\begin{array}{*{20}l} {\frac{\lambda }{n + \lambda } + (1 - \alpha^{2} + \beta )} \hfill \\ {\frac{1}{2(n + \lambda )}} \hfill \\ \end{array} } \right.\begin{array}{*{20}l} {,k = 0} \hfill \\ {,k = 1\sim 2n} \hfill \\ \end{array} $$

where $\omega_{s}^{(k)}$ is the weight of the sigma point mean, $\omega_{c}^{(k)}$ is the weight of the sigma point covariance. The parameter $\lambda { = }\alpha^{2} \left( {n + \kappa } \right) - n$ enables scaling of the weights to control the prediction error of the nonlinear system. $\alpha$ is the parameter that implements the control of the state of the sigma point distribution. $\beta$ is the perturbation error used to combine the higher order terms in the equation. $\kappa$ is the parameter that guarantees the semi-positive determination of the covariance matrix.

For the sake of discussion, the 2*n* + 1 sigma points are expressed in the form of Eq. (8).

8 $$ X_{(i|i)}^{(k)} = [\begin{array}{*{20}c} {\mathop X\limits^{ \wedge }_{(i|i)} } & {\mathop {,X}\limits^{ \wedge }_{(i|i)} + \sqrt {(n + \lambda )P_{(i|i)} } } & {\mathop {,X}\limits^{ \wedge }_{(i|i)} - \sqrt {(n + \lambda )P_{(i|i)} } } \\ \end{array} ] $$

where $\overline{X}_{(i|i)}$ is the evaluated value of the sigma point set at moment *i*.

One of the key steps of the prediction-stage Kalman filter. The predicted state evaluation value $X_{{\left( {i + 1|i} \right)}}^{\left( k \right)}$ at the *k*th sigma point is the predicted value based on the state at the previous moment, as shown in Eq. (9).

9 $$ X_{(i + 1|i)}^{(k)} = f(i,X_{(i|i)}^{(k)} ) $$

The system state prediction with covariance for the traceless Kalman filter is obtained by weighted averaging of the predicted values of the set of 2*n* + 1 sigma points. As shown in Eq. (10) and Eq. (11).

10 $$ \mathop X\limits^{ \wedge }_{(i + 1|i)} = \sum\limits_{k = 0}^{2n} {\omega_{s}^{(k)} X_{(i + 1|i)}^{(k)} } $$

11 $$ P_{(i + 1|i)} = \sum\limits_{k = 0}^{2n} {\omega_{c}^{(k)} \mathop {[X}\limits^{ \wedge }_{(i + 1|i)} - X_{(i + 1|i)}^{(k)} ]\mathop {[X}\limits^{ \wedge }_{(i + 1|i)} - X_{(i + 1|i)}^{(k)} ]^{{\text{T}}} + Q} $$

where *Q* is the covariance matrix of the input noise *W*_(*i*)_.

The correction phase is another key step in the Kalman filter. The predicted values are corrected and predicted according to the collected values to continuously approximate the true values. This phase starts with the unscented transformation of the predicted value of the system state at moment *i* + 1, as shown in Eq. (12).

12 $$ X_{(i + 1|i)}^{(k)} = [\begin{array}{*{20}c} {\mathop X\limits^{ \wedge }_{(i + 1|i)} } & {\mathop {,X}\limits^{ \wedge }_{(i + 1|i)} + \sqrt {(n + \lambda )P_{(i + 1|i)} } } & {\mathop {,X}\limits^{ \wedge }_{(i + 1|i)} - \sqrt {(n + \lambda )P_{(i + 1|i)} } } \\ \end{array} ] $$

The observed evaluation value $Z_{(i + 1|i)}^{(k)}$ for the *k*th sigma point, as shown in Eq. (13).

13 $$ Z_{(i + 1|i)}^{(k)} = g[X_{(i + 1|i)}^{(k)} ] $$

Based on the observed evaluated values of 2*n* + 1 sigma points, the weighted average gives the system prediction mean and covariance, which are Eqs. (14) to (15), respectively.

14 $$ \mathop Z\limits^{ \wedge }_{(i + 1|i)} = \sum\limits_{k = 0}^{2n} {\omega_{s}^{(k)} Z_{(i + 1|i)}^{(k)} } $$

15 $$ P_{{Z_{i} Z_{i} }} = \sum\limits_{k = 0}^{2n} {\omega_{c}^{(k)} [Z_{(i + 1|i)}^{(k)} - \mathop Z\limits^{ \wedge }_{(i + 1|i)} ][Z_{(i + 1|i)}^{(k)} - \mathop Z\limits^{ \wedge }_{(i + 1|i)} ]^{{\text{T}}} + R} $$

16 $$ P_{{X_{i} Z_{i} }} = \sum\limits_{k = 0}^{2n} {\omega_{c}^{(k)} [X_{(i + 1|i)}^{(k)} - \mathop X\limits^{ \wedge }_{(i + 1|i)} ][Z_{(i + 1|i)}^{(k)} - \mathop Z\limits^{ \wedge }_{(i + 1|i)} ]^{{\text{T}}} } $$

The Kalman gain can be calculated from the correlation coefficient $P_{{X_{i} Z_{i} }}$ between the state and the observation and the observation variance $P_{{Z_{i} Z_{i} }}$, as shown in Eq. (17).

17 $$ K_{(i + 1)} = P_{{X_{i} Z_{i} }} P_{{Z_{i} Z_{i} }}^{ - 1} $$

By correcting the predicted data at moment *i* + 1, the system state and covariance are obtained as Eqs. (18) and (19), respectively.

18 $$ \mathop X\limits^{ \wedge }_{(i + 1|i + 1)} = \mathop X\limits^{ \wedge }_{(i + 1|i)} + K_{(i + 1)} [Z_{(i + 1)} - \mathop Z\limits^{ \wedge }_{(i + 1|i)} ] $$

19 $$ P_{(i + 1|i + 1)} = P_{(i + 1|i)} - K_{(i + 1)} P_{{Z_{i} Z_{i} }} K_{(i + 1)}^{{\text{T}}} $$

**Figure 7:**
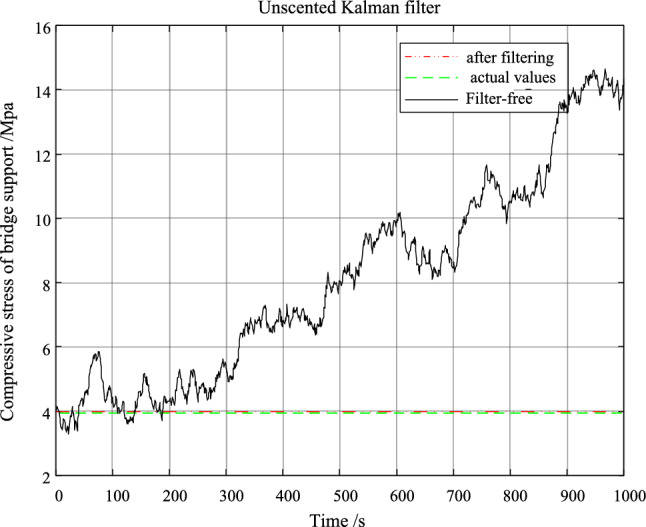
Simulation result diagram of unscented Kalman filter.

The performance of the unscented Kalman filter in this system is verified by MATLAB simulation, the set algorithm parameters are: $\alpha { = }1$, $\beta { = }2$, $\kappa { = }2$. The simulation results are shown in Fig. 7.

**Figure. 8:**
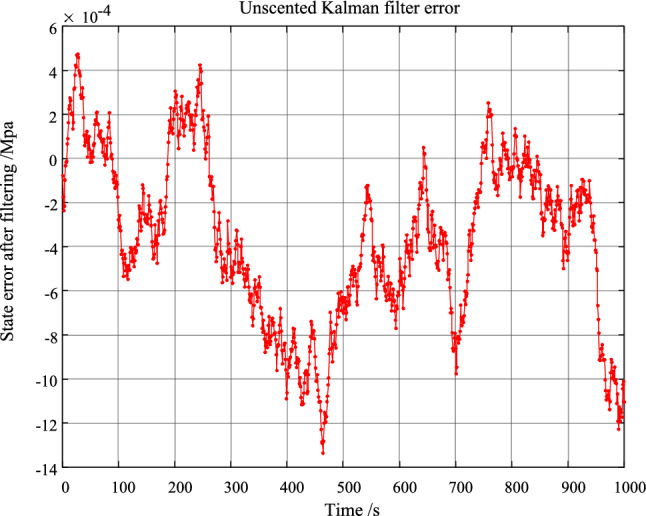
Error result chart of unscented Kalman filter.

According to Fig. 7, it can be seen that the signal containing noise can be well filtered out by unscented Kalman filter. Moreover, the filtering result is very close to the real signal. In order to accurately know the filtering effect of unscented Kalman filter, the filtering error is given in Fig. 8.

According to Fig. 8, the unscented Kalman filter can reduce the noise error by 4 orders of magnitude. The monitoring accuracy of the system can be improved.

### Monitoring server

JAVA is chosen as the development language of monitoring server, Alibaba Cloud server as the remote bridge monitoring server, and physical server to build data forwarding server at the bridge site. Firstly, the cloud server is configured with Windows Sever image, and then this image is used to develop the Web client. The collected compressive stress data information is dynamically and statically displayed on the Web. If the compressive stress signal exceeds the threshold, the alarm information is sent to the relevant responsible person.

**Figure 9:**
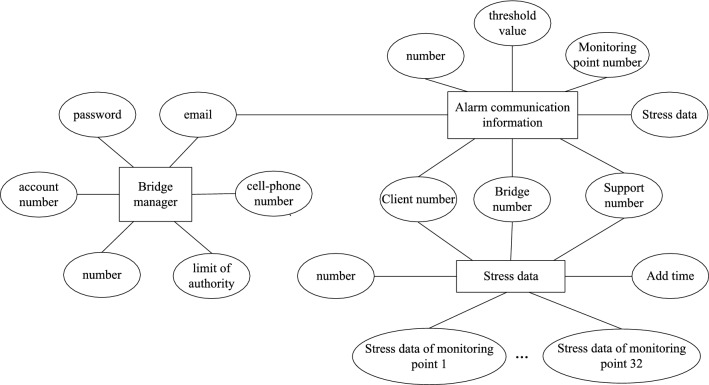
E–R diagram of database.

As the carrier of bridge bearing stress data and bridge management platform related information, the database needs to provide safe storage function and efficient data reading and writing function. The database software is arranged on the cloud server, and accesses MySQL database through the dedicated database access interface through the network monitoring server, communication server, web-side bridge management platform, etc. According to the functional requirements of the system, the data objects stored in the database are divided into bridge managers, alarm communication information and stress data. Fig. 9 shows the E–R diagram of the database.

When the stress data of the bridge support exceeds the warning threshold set by the manager, the real-time alarm program will be triggered. The real-time alarm function is set to mail alarm, monitoring interface alarm and mobile client alarm. When the compressive stress exceeds the threshold value, the information such as the current stress value will be sent to the bridge manager by email. The interface of the monitoring platform and the mobile client will be marked simultaneously.

## Test and result

### Sensor testing and calibration

**Figure 10:**
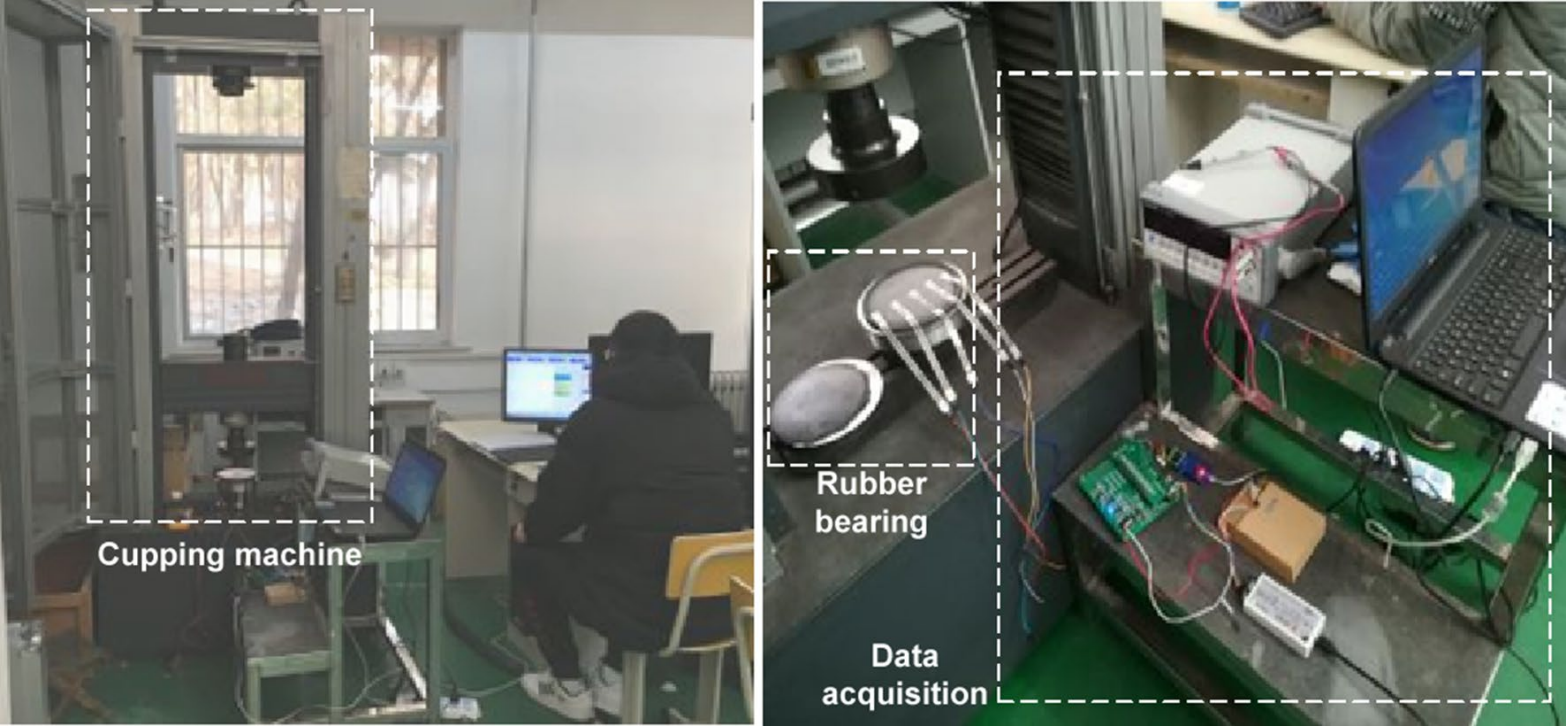
Loading and unloading experimental environment of bridge bearing monitoring system.

In order to validate the feasibility and effectiveness of the proposed system, a bridge bearing compressive stress test platform is built. Due to the limitation of the column spacing of the tensile pressure testing machine, the actual bridge bearing size cannot meet the testing space of the testing machine. Therefore, a simulated basin bearing with a diameter of 17 cm is processed to build up the test environment. The bearing has a 150 mm rubber plate and a 160 mm diameter steel cover plate. The experimental environment is shown in Fig. 10.

The test operation steps are as follows:

1. A thin film pressure sensor is arranged in the middle of the upper plane of the rubber plate, a thin rubber layer is laid flat on the surface of the sensor, and then a steel cover is placed on the top layer of the rubber as a direct bearing point.
2. The tester is loaded on the support, i.e. from 0 to 6 MPa slowly, and in order to obtain more data under different compressive stress levels, the tester is set to last for 1 min for every 0.5 MPa increase in compressive stress, and is recorded and displayed by computer software.
3. Unloading operation after the tester loads the support to the maximum value, i.e. from 6 to 0 MPa slowly, in order to obtain more data under different compressive stress levels, set the tester to continue for 1 min for each 0.5 MPa reduction in compressive stress, and record the collected data displayed.
4. If you need to repeat the test for the same sensor, repeat steps (2) to (3); for different sensors, repeat steps (1) to (3).

**Figure 11:**
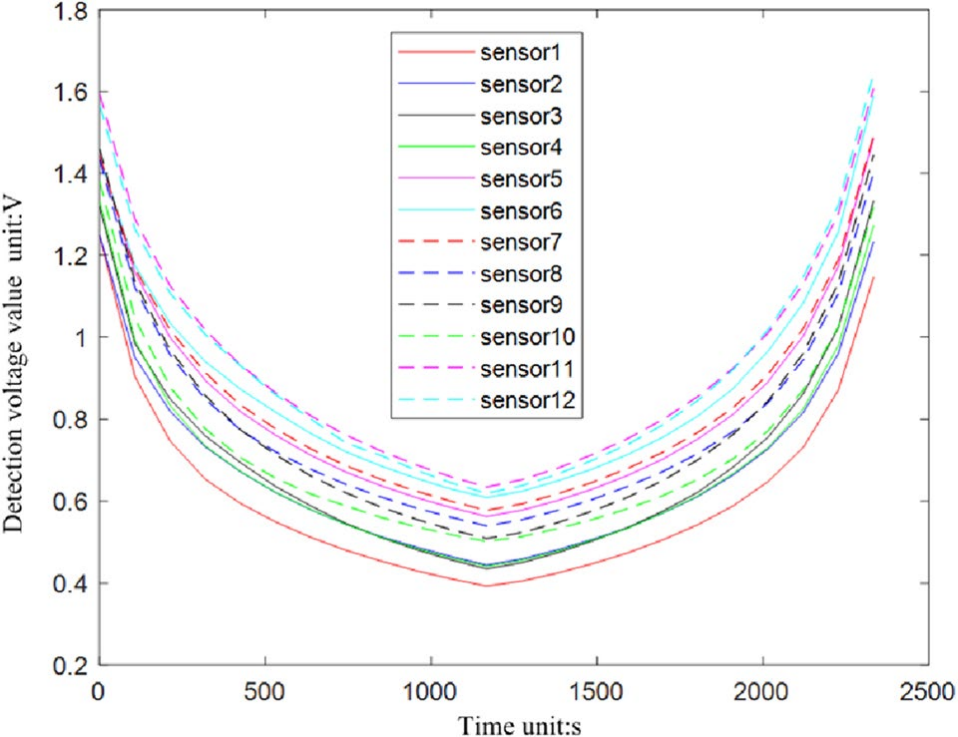
Sensor suitability test results.

In order to verify the applicability of sensor technical indexes to the stress detection of bridge supports, five tests were conducted on 12 sensors respectively, and averaged the results of five tests on each sensor, as shown in Fig. 11.

According to the test, the test repeatability of all 12 sensors is within ± 3.2%, and most of the sensor repeatability values are within ± 2%, so the sensor has good repeatability index to meet the demand of bridge bearing compressive stress monitoring. However, for the sensor linearity index, the measurement voltage magnitude of different sensors under uniform applied pressure varies greatly, i.e., the consistency of the sensors is poor, which cannot meet the demand of high precision monitoring.The system is based on the traditional least squares fitting^32–34^ to improve the second-order curve fitting based on least squares.

Because Flexiforce A201 sensor has good repeatability, the following calibration of the sensor will take the loading data of compressive stress as the analysis object. Each sensor repeatedly collects five times of pressurization, and each loading operation collects data of different compressive stresses for many times. Average the data collected in five tests under the same compressive stress, and curve fitting the average values under different compressive stresses.

**Figure 12:**
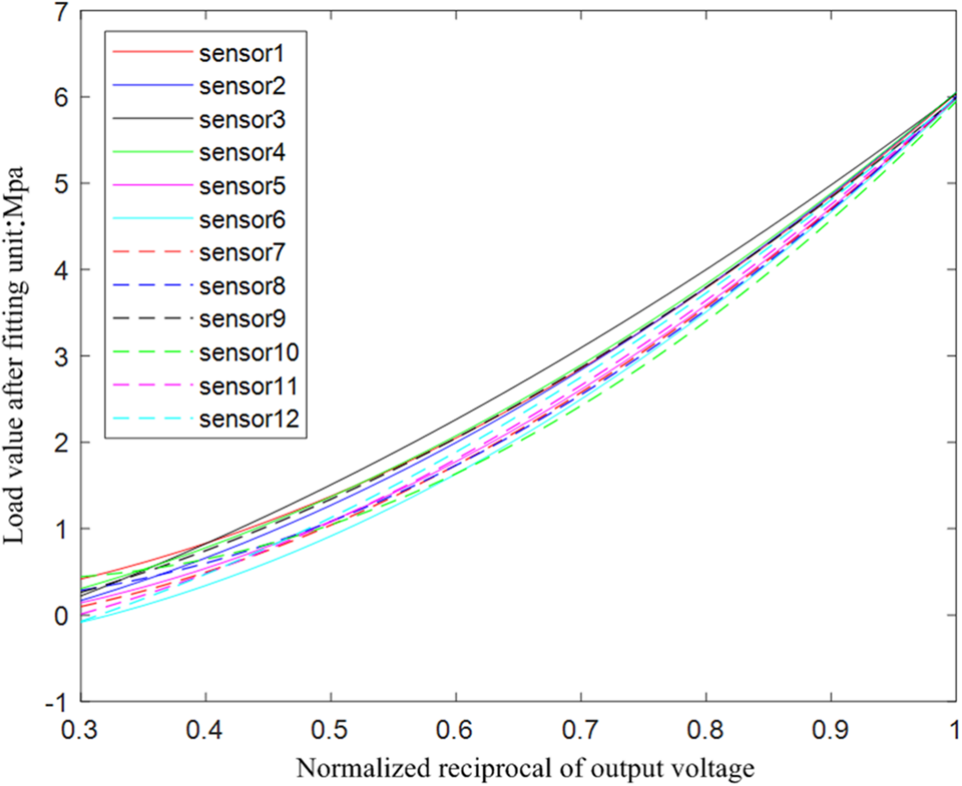
Sensor calibration fitting curve test result.

According to the principle of sensor calibration, the inverse parameter $1/\delta_{i}$ is taken from the mean value of the direct output voltage $u_{ij}$. And then normalized to obtain the parameter $x_{i}$. Finally, the parameter $x_{i}$ is fitted with the load $y_{i}$ by the least square method. The fitted curve is shown in Fig. 12, which shows that the data accuracy has been improved.

The non-linear error is used to further measure the linearity index of the accurate analysis sensor. The non-linear error after calibration and the polynomial used for fitting are shown in Table 3. The data show that the calibrated sensor has a good linearity index, which meets the demand for accurate monitoring of the compressive stress of the bridge support.

**Table 3:** Sensor calibration data.

Sensor number	Fitted polynomial	Non-linear error (%)
1	$y = \frac{0.9969}{{u^{2} }} - \frac{0.1502}{u} - 0.0505$	± 1.6
2	$y = \frac{1.1380}{{u^{2} }} + \frac{0.3994}{u} - 0.6212$	± 2.2
3	$y = \frac{0.7054}{{u^{2} }} + \frac{1.4983}{u} - 1.1488$	± 3.2
4	$y = \frac{1.1229}{{u^{2} }} + \frac{0.3056}{u} - 0.4223$	± 2.3
5	$y = \frac{2.2962}{{u^{2} }} - \frac{0.6050}{u} - 0.1877$	± 0.6
6	$y = \frac{2.7054}{{u^{2} }} - \frac{0.5086}{u} - 0.4904$	± 2.0
7	$y = \frac{2.4638}{{u^{2} }} - \frac{0.6870}{u} - 0.2097$	± 0.5
8	$y = \frac{2.4344}{{u^{2} }} - \frac{1.4569}{u} + 0.3441$	± 0.8
9	$y = \frac{1.4417}{{u^{2} }} + \frac{0.4771}{u} - 0.5191$	± 0.6
10	$y = \frac{2.4431}{{u^{2} }} - \frac{2.3853}{u} + 0.9970$	± 1.7
11	$y = \frac{2.5811}{{u^{2} }} + \frac{0.1366}{u} - 0.6303$	± 1.0
12	$y = \frac{2.0425}{{u^{2} }} + \frac{1.0796}{u} - 1.0750$	± 2.7

### Data acquisition card performance analysis

The accuracy analysis of the acquisition board in this paper is mainly divided into two aspects: the zero-input response and the 1/2 range-input response.

When dealing with the input short circuit of the acquisition card, the zero-input response is mainly determined by the characteristics of the card itself and the initial state of the card. When the acquisition card is working at zero time, it can be determined by testing that it has little influence on zero-input response. Therefore, the analysis of zero-input response to the acquisition card is an analysis of the noise performance to the acquisition card itself.

**Figure 13:**
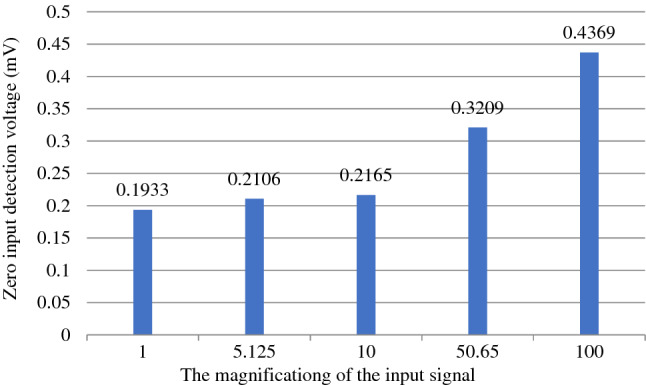
Zero-input response to the acquisition card.

Firstly, we short-circuit the input terminals of the data acquisition card with different magnifications to produce a zero-input response that is mainly determined by the characteristics of the board itself. The result is shown in Fig. 13. When the amplification of the acquisition card with zero-input detection voltage is 1x, the noise is the smallest, which is 193 μV. Because the linear error accuracy of the sensors of the bridge support monitoring system is about plus or minus 3%, the noise error of the acquisition board is much lower than the sensor detection error. Thus, the accuracy of the acquisition board can fully meet the system requirements.

The maximum input signal to the acquisition system must be 1/2 to 2/3 of the full scale input to ensure accurate signal acquisition and processing by the system. Therefore, in this paper, the input response within 1/2 range is analyzed to determine the noise immunity of the acquisition board to the input signal from both the mean and variance of the output signal to ensure the high accuracy requirements of the acquisition board.

**Figure 14:**
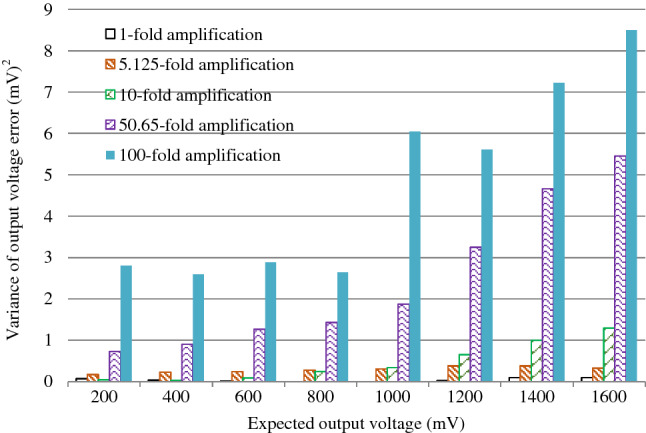
Analysis graph of input response variance within 1/2 ran.

To determine the accuracy of the acquisition board's information acquisition, this test uses a Keithley seven-and-a-half digit precision multimeter to perform comparison tests on the signals. First, the signals with different input amplitudes of the acquisition board were measured by a high-precision multimeter. Then the magnitude of the output voltage of the acquisition board is calculated according to the amplification of the acquisition board. At the same time, the acquisition board is made to perform multiple data acquisition for signals with different input amplitudes. Finally, the average value of the output voltage value and the detection voltage of the acquisition board are compared and calculated, and the test data are shown in Table 4. The variance of the voltage collected by the data acquisition card and the meter are calculated respectively. The result is shown in Fig. 14.

**Table 4:** Comparison of instrument measurement and board detection output voltage.

Attributes		Expected output voltage(mV)							
		200	400	600	800	1000	1200	1400	1600
Onefold amplification(mV)	Measurements	201.2	401.4	601.4	801.9	1001.9	1201.4	1401.5	1601.2
	Theoretical values	201.2	401.4	601.4	801.9	1001.9	1201.4	1401.5	1601.2
	Collected values	201.0	401.2	601.3	801.8	1002.0	1201.6	1401.8	1601.5
5.125-fold amplification(mV)	Measurements	40.70	80.98	120.85	161.15	201.24	240.94	280.61	320.73
	Theoretical values	208.6	415.0	619.4	825.9	1031.4	123.80	1438.1	1643.7
	Collected values	334.7	414.6	618.9	825.4	1030.8	1234.2	1437.5	1643.2
tenfold amplification(mV)	Measurements	20.65	40.71	60.62	80.98	101.15	120.86	141.02	161.15
	Theoretical values	206.5	407.1	606.2	809.8	1011.5	1208.6	1410.2	1611.5
	Collected values	206.4	407.1	606.4	810.3	1012.1	1209.4	1411.2	1612.6
50.56-foldamplification(mV)	Measurements	4.590	8.500	12.50	16.58	20.740	24.810	28.770	32.720
	Theoretical values	232.5	430.5	633.1	839.8	1050.5	1256.6	1457.2	1657.3
	Collected values	232.7	431.0	633.9	840.6	1051.6	1258.2	1459.2	1659.5
100-fold amplification(mV)	Measurements	4.590	8.500	12.50	16.58	20.740	24.810	28.770	32.720
	Theoretical values	232.5	430.5	633.1	839.8	1050.5	1256.6	1457.2	1657.3
	Collected values	232.7	431.0	633.9	840.6	1051.6	1258.2	1459.2	1659.5

Table 3 shows that under different magnifications, the average of the output voltage measured by the multimeter and the average value of the detection voltage of the acquisition card are very close. This indicates the proposed system can meet the requirements of accurate monitoring. As shown in Fig. 14, under the condition of magnification ≤ 10, the variance between the output voltage value measured by the high-precision meter and the output voltage value of the acquisition card is generally less than 1mV^2^, which can fully meet the requirements of the high-precision monitoring system demand.

### Verification feasibility experiment

To verify the feasibility of the system, a 300kN tensile tester is used to perform the axial compression stress test and the bias compression test respectively on the calibrated bridge support monitoring system. For different sensors, the consistency is different. In order to reduce this difference, the method of relative detection is adopted. The detection change of each sensor is obtained to determine the local compressive stress change of the support, by comparing the axial pressure and bias pressure detection data.

**Figure 15:**
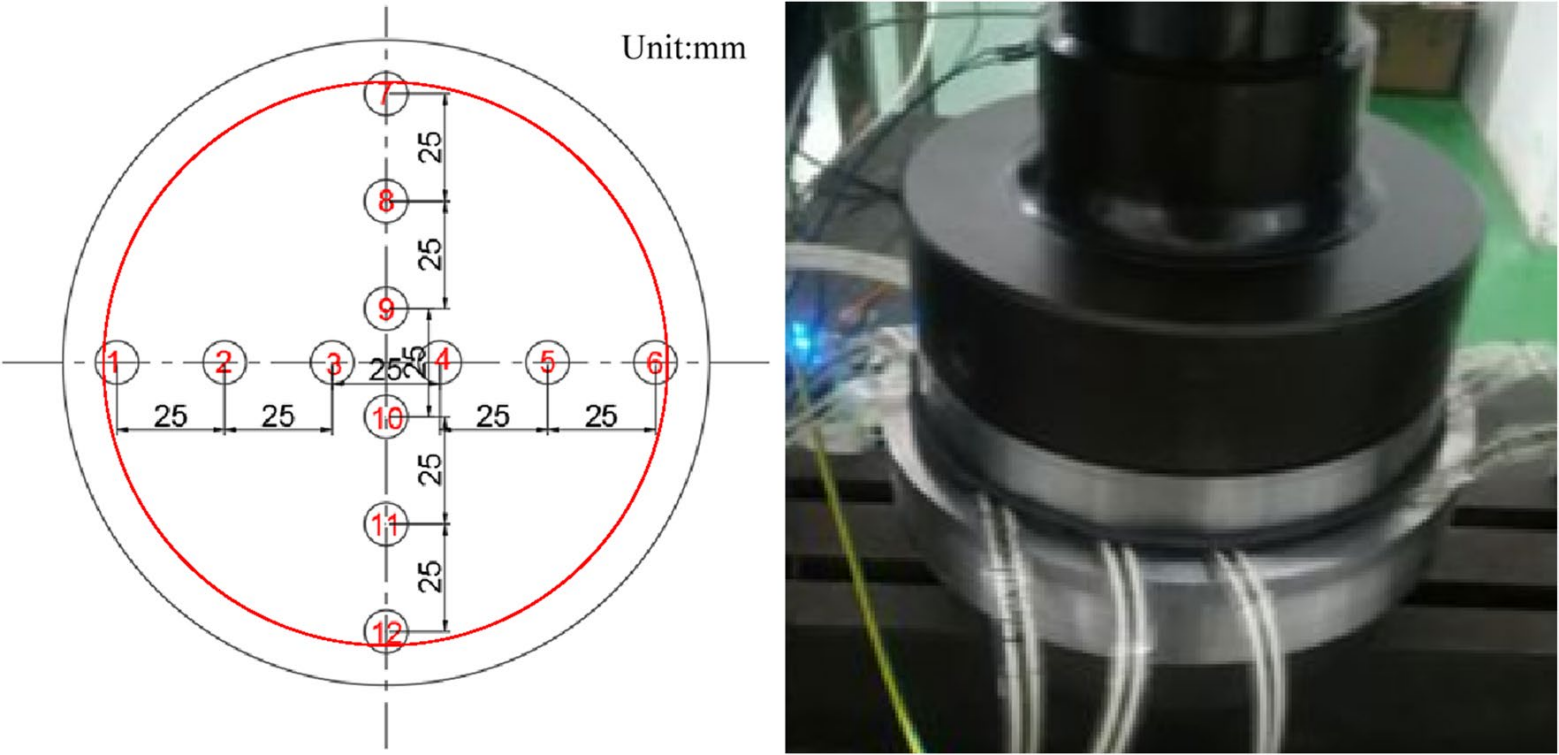
Sensor layout scheme and physical drawing of axial bearing.

As shown in Fig. 15, it is the sensor layout scheme and physical drawing for axial loading test. Sensors 1 to 6 are placed along one diameter horizontally, and sensors 7 to 12 are placed along the other diameter vertically. The distance between the two sensors is set to 25 mm.

**Figure 16:**
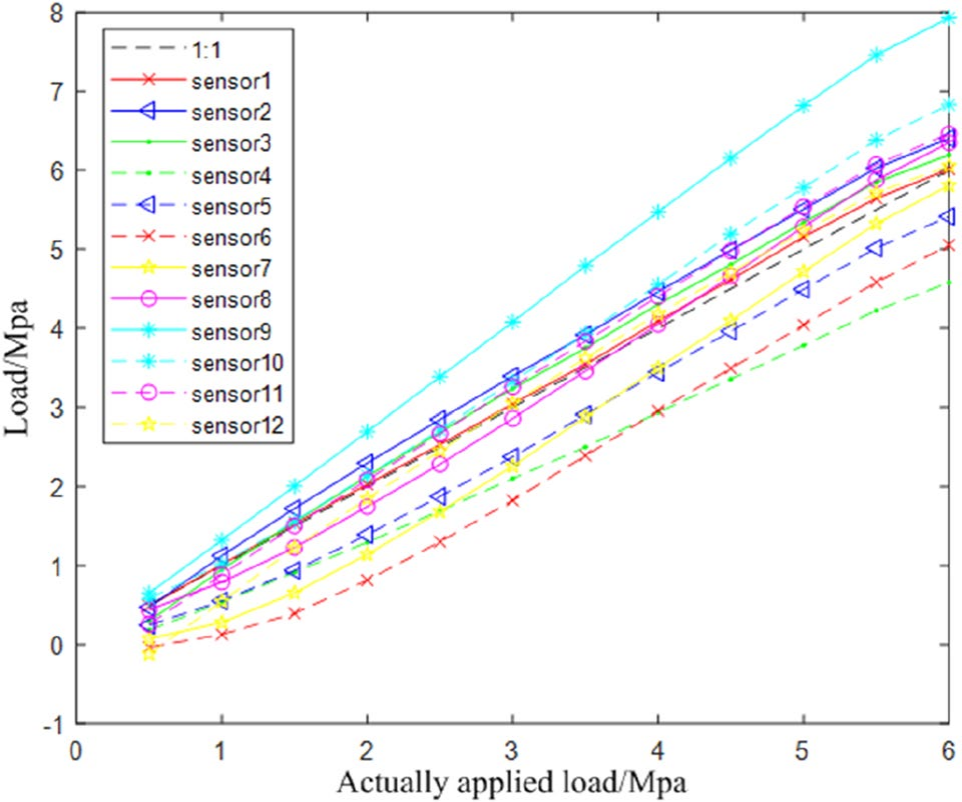
Relationship curve between applied load and detected load of axial pressure.

The relationship between the applied load and the test load in axial compression loading test is shown in Fig. 16, in which it can be clearly observed that the relationship between the ratio of the applied load and the detected load of each sensor is close to 1:1. And the linearity of the relationship curve of a single sensor is high. However, due to the slight differences between different sensors and the unpredictability of the extruding deformation of the bearing rubber, the absolute detection amount of multiple sensors is different.

**Figure 17:**
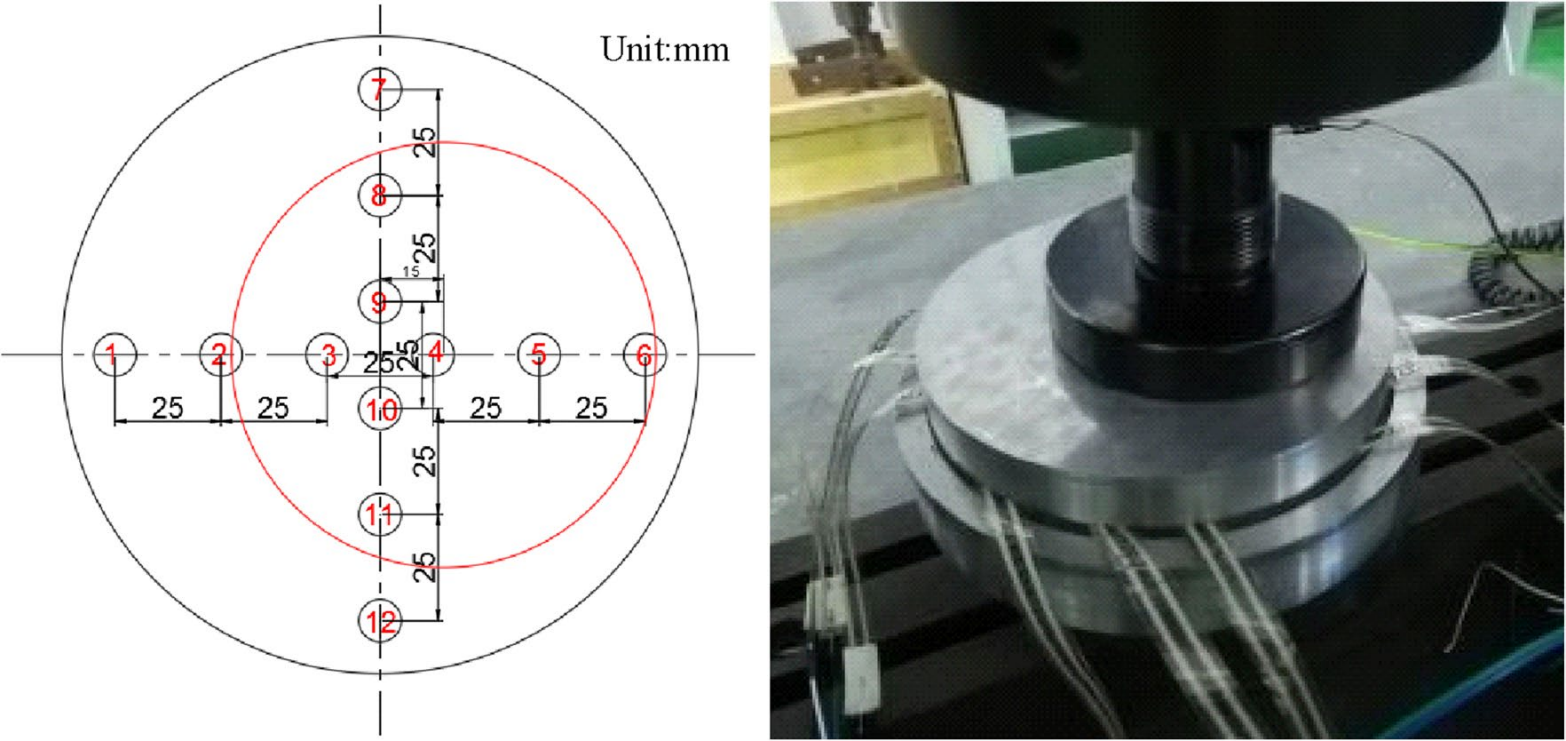
Sensor layout scheme and physical drawing of bias bearing.

**Figure 18:**
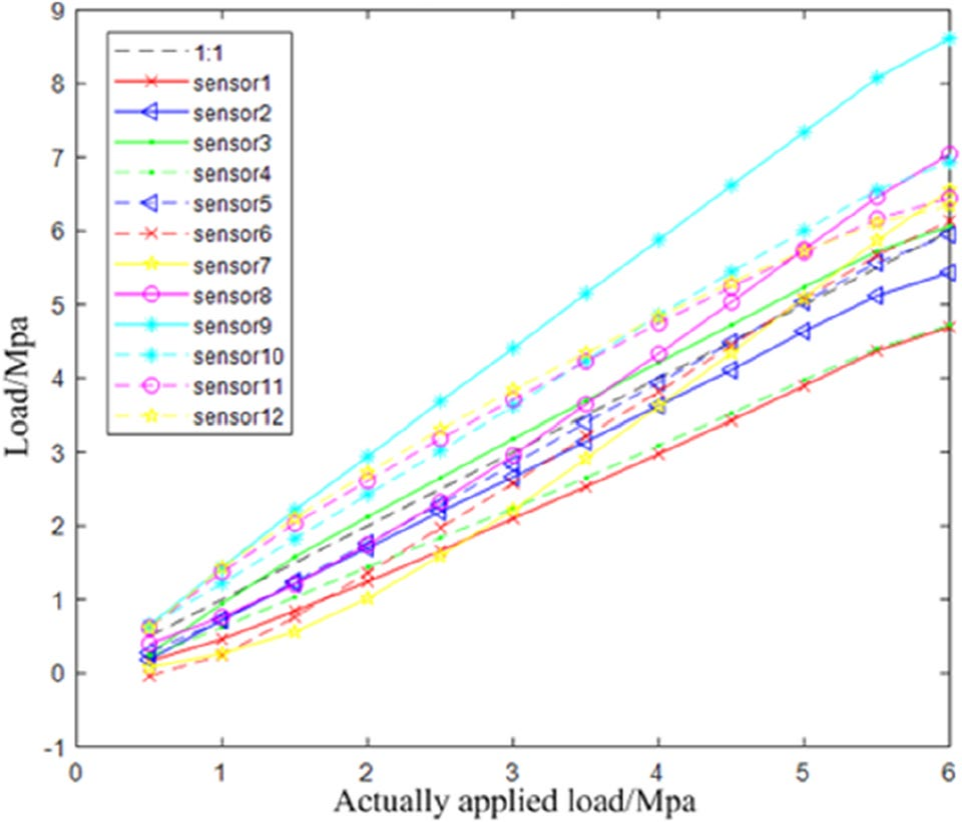
Relationship curve between applied load and detected load of bias pressure.

Bias loading test is shown in Fig. 17. The sensor arrangement is the same as that of axial compression loading test. Apply a load to the point off the axis of the bearing. The relationship between the applied load and the test load in axial compression loading test is shown in Fig. 18. Compared with the axial compression curve of Fig. 16, it can be seen that the linear slope of the relationship of each sensor change.

**Figure 19:**
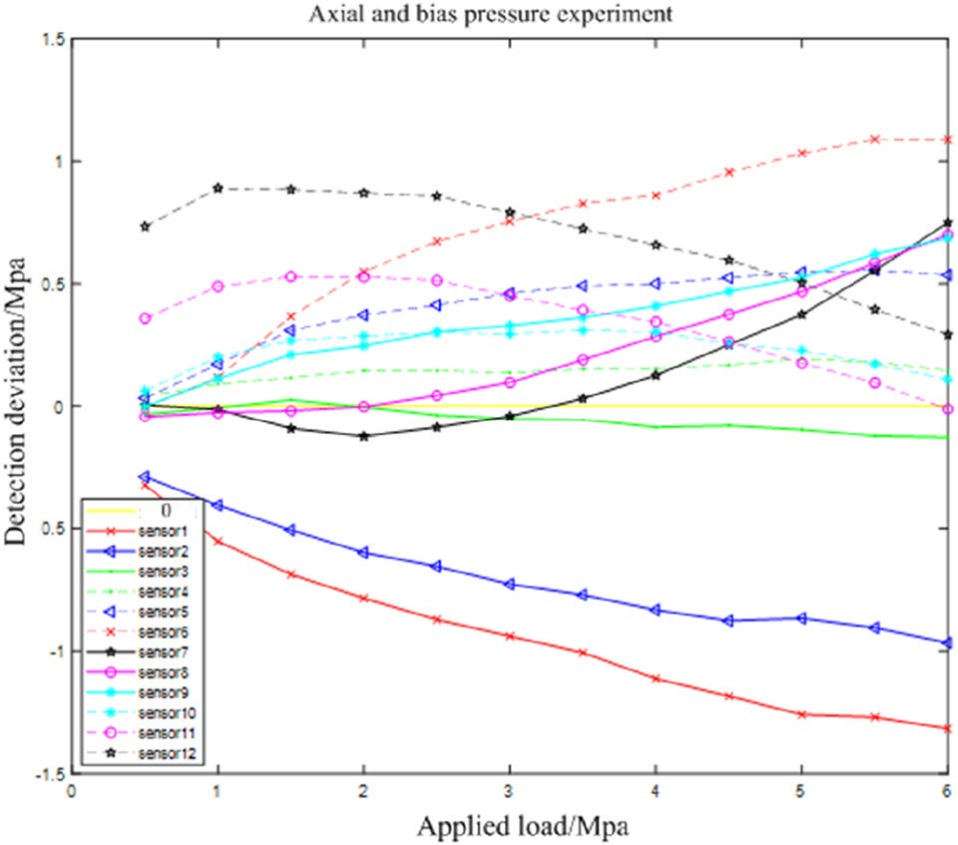
Correspondence graph between bias load and applied load for bias detection.

Under different bias and axial pressure conditions, the local force of the stent is different, and it is impossible to accurately determine the local force of the support. Thus, the relationship between applied and detected load under axial pressure is used as a reference to explore the relative load value detected under biased conditions. Under the same applied load of the same sensor, the bias detection load $fb$ is subtracted from the axial pressure detection load $fa$, i.e., the bias detection deviation load $\Delta f$, to obtain the relative deviation value. The relationship between the applied and the detected deviation load is shown in Fig. 19.

**Figure 20:**
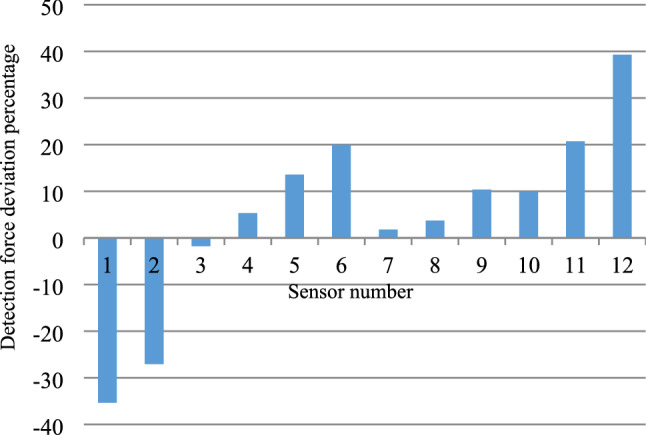
Test result graph of detection force deviation percentage of sensors.

According to the situation that the sensor detected the deviation load deviates from zero in Fig. 19, the force change of the sensor under the bias pressure condition can be roughly determined. However, Fig. 19 cannot clearly show the local force of the bearing. In order to accurately and clearly analyze the force of the bearing, detected pressure offset percentage variable $\omega = \frac{\Delta f}{{fa}}$ is introduced. The detection pressure deviation percentages under different applied loads of the same sensor are accumulated to obtain the average value of the detection force deviation percentages of each sensor under different pressure conditions, as shown in Fig. 20.

According to Fig. 20, the force is the largest in the area of the 90° support where the sensors 10–12 and 4–6 are located, and the force is the smallest in the area of the 90° support where sensors 1–3 and 7–9 are located. Fig. 20 shows that the stress conditions are consistent with the sensor bias conditions of Fig. 17 . Therefore, the bridge support compressive stress monitoring system is feasible.

### Testing of monitoring server

**Figure 21:**
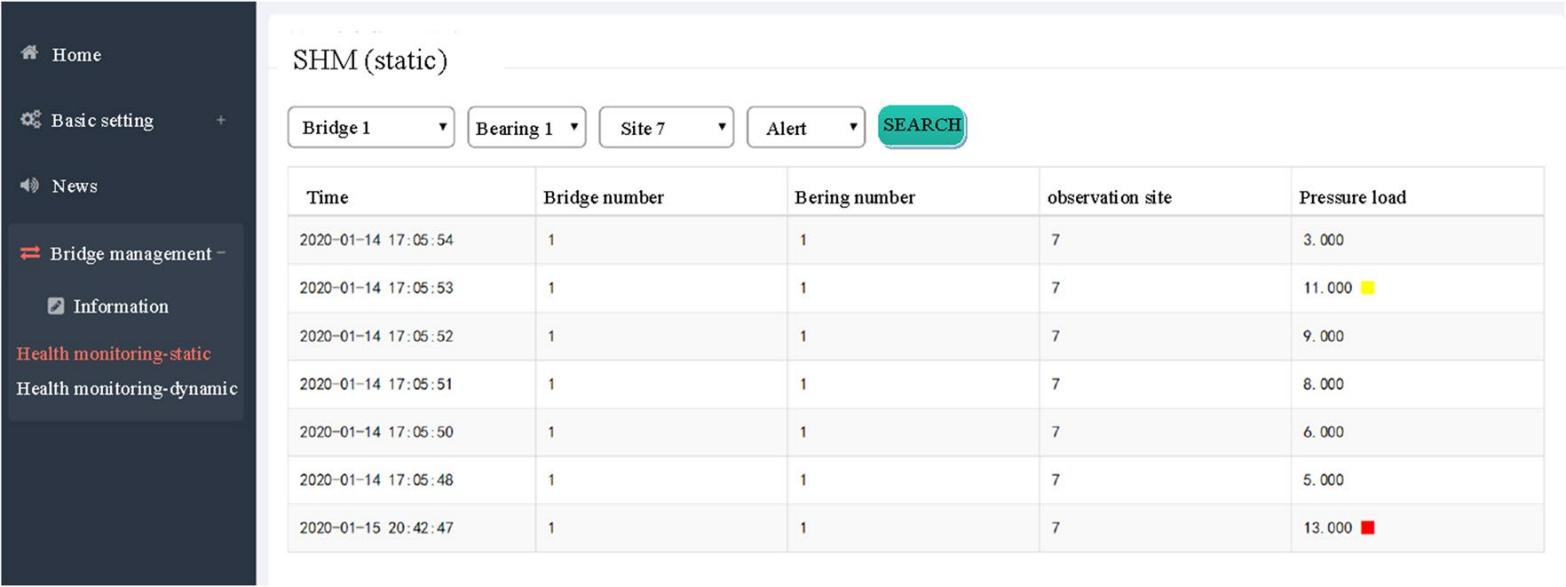
Bridge health monitoring static display interface.

The real-time monitoring of the bridge bearing stress data is shown in Fig. 21. When the compressive stress data exceeds the threshold value, a Class I yellow warning or a Class II red warning is displayed in the Pressure Load column.

## Conclusion

The health monitoring system of bridge bearing should accurately obtain the internal stress information, and then judge and analyze the health status of the bridge. According to the application requirements of high-precision real-time monitoring of bridge bearings, the health monitoring system is designed. With the help of bridge bearing loading tests, the sensors calibration and applicability verification is carried out. And evaluate the accuracy of the acquisition board is evaluated and the overall feasibility of the force monitoring system is analyzed. According to the actual detection results, the detection error of the acquisition board is less than 200 μV, which can realize the complementary application of multichannel sensor signal acquisition and multiple communication methods. The accuracy of the calibrated sensor is around ± 1.6%. The system can accurately determine stress distribution, and meets the needs of bridge support health monitoring. This monitoring system is suitable for new or rehabilitated bearings since the sensors to measure the internal stress need be embedded in the bearings. In future research, the bridge management system based on long-term monitoring, early warning and project management will be studied. Improve the versatility of the bridge monitoring system based on existing monitoring programs and actual operation modes.

## Data Availability

Te datasets generated during and/or analysed during the current study are available from the corresponding author on reasonable request. Contact person: xncao@mail.sdu.edu.cn.
